# Boosting 2000‐Fold Hypergolic Ignition Rate of Carborane by Substitutes Migration in Metal Clusters

**DOI:** 10.1002/advs.202401861

**Published:** 2024-04-03

**Authors:** Jia‐Hong Huang, Ao‐Qi Ji, Zhao‐Yang Wang, Qian‐You Wang, Shuang‐Quan Zang

**Affiliations:** ^1^ Henan Key Laboratory of Crystalline Molecular Functional Materials, and College of Chemistry Zhengzhou University Zhengzhou 450001 China

**Keywords:** copper clusters, energetic materials, hypergolic fuels, metal clusters

## Abstract

Hypergolic propellants rely on fuel and oxidizer that spontaneously ignite upon contact, which fulfill a wide variety of mission roles in launch vehicles and spacecraft. Energy‐rich carboranes are promising hypergolic fuels, but triggering their energy release is quite difficult because of their ultrastable aromatic cage structure. To steer the development of carborane‐based high‐performance hypergolic material, carboranylthiolated compounds integrated with atomically precise copper clusters are presented, yielding two distinct isomers, Cu_14B‐S_ and Cu_14C‐S_, both possessing similar ligands and core structures. With the migration of thiolate groups from carbon atoms to boron atoms, the ignition delay (ID) time shortened from 6870 to 3 ms when contacted with environmentally benign oxidizer high‐test peroxide (HTP, with a H_2_O_2_ concentration of 90%). The extraordinarily short ignition ID time of Cu_14B‐S_ is ranking among the best of HTP‐active hypergolic materials. The experimental and theoretical findings reveal that benefitting from the migration of thiolate groups, Cu_14B‐S_, characterized by an electron‐rich metal kernel, displays enhanced reducibility and superior charge transfer efficiency. This results in exceptional activation rates with HTP, consequently inducing carborane combustion and the simultaneous release of energy. This fundamental investigation shed light on the development of advanced green hypergolic propulsion systems.

## Graphical abstract

Two carboranylthiolate‐protected isometric copper clusters (Cu_14B‐S_ and Cu_14C‐S_) show distinctly different ID times (three orders of magnitude difference) upon environmentally benign oxidizer high‐test peroxide (HTP) ignition. Based on the isomeric platform, the significantly boosted ignition rate with HTP is attributed to their distinct electronic structure, which leads to different activation rate toward triggering carborane “open face” and release of energy.

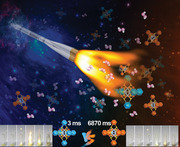

## 1 Introduction

**Figure 1:**
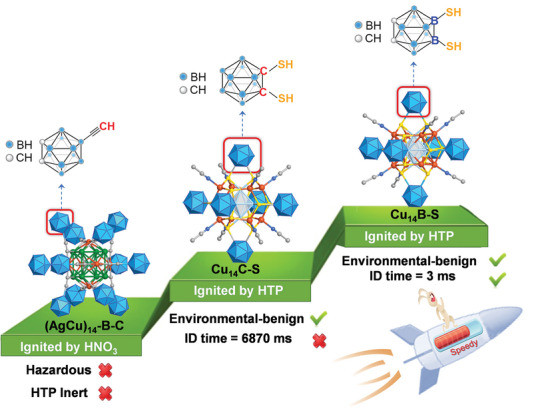
Comparison of the hypergolic properties of 9‐HC≡C‐*o*‐carborane,^[^ ^29^ ^]^ 1,2‐(HS)_2_‐*o*‐carborane, and 9,12‐dimercapto‐(HS)_2_‐*o*‐carborane‐protected metal clusters.

Energetic materials, capable of storing and releasing substantial chemical energy on demand, are vital in material science.^[^ ^1^, ^2^, ^3^, ^4^, ^5^ ^]^ Hypergolic materials exemplify these compounds, igniting spontaneously upon contact with specific oxidizers.^[^ ^6^, ^7^, ^8^, ^9^ ^]^ The combination of hypergolic fuel and oxidizer is termed bipropellant, which is the power source of propulsion systems in rockets or spacecraft.^[^ ^10^, ^11^, ^12^, ^13^, ^14^ ^]^ Boron could be a potential solid fuel for this application due to high gravimetric (≈60 MJ kg^−1^) and volumetric (≈140 MJ m^−3^) heats of reactions.^[^ ^15^, ^16^, ^17^ ^]^ In recent years, studies of borohydride and periodoborane clusters as hypergolic fuels have been reported.^[^ ^18^ ^]^ However, their practical implementation is hindered by poor thermal and water‐stability, and inferior compatibility. Carboranes (icosahedral *closo*‐carboranes) are a class of carbon‐boron molecular clusters with a 3D aromatic cage structure. Benefitting from its steric hindrance and fine‐tuned structure, carboranes have been widely used as a building block for the construction of photofunctional materials, catalysts, and drugs.^[^ ^19^, ^20^, ^21^, ^22^ ^]^ Integrating the remarkable stability, high boron content, and high output energy (Δ*H*_f_ = 42 kcal mol^−1^),^[^ ^23^, ^24^, ^25^, ^26^, ^27^, ^28^ ^]^ carborane can aid in compensating for the difficulties associated with borohydride fuels, but they are insensitivity toward oxidizers and the so‐called stability‐activity trade‐off. To address this challenge, we recently reported that constructing carboranealkynyl ligands‐protected metal clusters with atomically precise structures, in which metal kernels function as catalytic sites could trigger hypergolic ignition of carboranes.^[^ ^29^ ^]^ The fusion of atomically engineered metal clusters with carboranes represents a promising strategy for inducing the spontaneous combustion of carboranes.^[^ ^30^, ^31^, ^32^, ^33^ ^]^ However, the relatively longer ignition delay time (140 ms for [Ag_14_(C_4_B_10_H_11_)_12_(CH_3_CN)_2_]·2NO_3_, 15 ms for [Cu_6_Ag_8_(C_4_B_10_H_11_)_12_Cl]NO_3_), defined as the time from the contact of the fuel with the surface of oxidizer to the first spark observed, remains unsatisfying for practical propulsion systems (**Figure** **1**). Additionally, using highly corrosive and carcinogenic white fuming nitric acid (WFNA) as an oxidizer is not favorable for the concept of green propulsion. The pursuit of environmentally friendly hypergolic bipropellants is gaining momentum.^[^ ^34^, ^35^, ^36^, ^37^ ^]^ In terms of oxidizers, high‐test peroxide (HTP) which contains highly concentrated non‐toxic H_2_O_2_ (>90%) is being pursued to replace the conventional WFNA.^[^ ^38^, ^39^, ^40^ ^]^ However, HTP showing relatively weak oxidation potential has limited its ignition capabilities.^[^ ^41^, ^42^ ^]^ Thus, how to steer hypergolic ignition of carboranes with HTP remains a significant challenge.

Copper nanoclusters possess atomically tunable structures, abundant active Cu sites, and high surface‐to‐volume ratios providing an ideal model system that can enable a fundamental investigation of the hypergolic ignition of carborane.^[^ ^43^, ^44^, ^45^, ^46^, ^47^, ^48^, ^49^, ^50^, ^51^, ^52^ ^]^ To enhance the combustion of carboranes with HTP, thiolate‐protected Cu nanoclusters are more attractive than alkynyl ligands‐based clusters because the weaker interactions of ‐SH unit to metal kernels relative to that of the C≡C bound to a metal atom lead to higher activity toward external oxidizer.^[^ ^53^, ^54^ ^]^ Besides, the introduction of a sulfur atom in carboranes may be conducive to ignition performance.^[^ ^55^, ^56^ ^]^ Based on the above considerations, we initially synthesized a carboranylthiolate‐protected superatomic copper‐cluster, Cu_14_(BC‐S)_6_(CH_3_CN)_8_ (BC‐S = 1,2‐dithiolate‐*o*‐carborane, abbreviated as Cu_14C‐S_), in which the dithiolate units attaching to the carbon atoms of carborane clusters. Upon contact with “rocket grade” HTP oxidizer, Cu_14C‐S_ demonstrates hypergolicty but an ultralong ID time of 6870 ms (Figure 1). Considering that the B atoms with less electronegative are more likely to give electrons than C atoms, the thiol functional groups migrate to boron vertices may provide opportunities for further enhancing the catalytic activity. Cu_14_(CB‐S)_6_(CH_3_CN)_6_ (CB‐S = 9,12‐dithiolate‐*o*‐carborane, abbreviated as Cu_14B‐S_), featuring similar core−shell geometry structure and valence electron counts but significantly different electronic structures with Cu_14C‐S_, is prepared. Cu_14B‐S_ exhibits a short ignition delay (ID) time of 3 ms, comparable to advanced hypergolic materials (Figure 1). Notably, metal complex isomers with such distinctly divergent hypergolic properties are rare. With the assistance of experimental and theoretical results, we elucidate that the migration of thiol groups in carboranes result in distinct activation energy to break the B─S and C─S bond toward Cu_14_, consequently triggering carborane “open face” and release of energy. This work not only breaks the challenging activity–stability trade‐off of carborane clusters as hypergolic fuel through systematically migrating the location of substitutes but also guides the future preparation of high‐performance and environmentally friendly metal cluster‐based hypergolic fuels.

## 2 Results and Discussion

### 2.1 Synthesis and Structural Characterization

Cu_14B‐S_ and Cu_14C‐S_ were synthesized through one‐pot reduction using borane *tert*‐butylamine, the detailed procedures were shown in Supporting Information (Figures S1 and S2, Supporting Information). The phase purity and elemental composition were characterized by powder X‐ray diffraction (PXRD) and energy‐dispersive spectrometry (EDS) measurements, respectively (Figures S3 and S4, Supporting Information). Fourier transform infrared (FT‐IR) spectra showed the characteristic band at 2594 cm^−1^ for B─H stretching and at 2304 and 2273 cm^−1^ for ─CN stretching (Figure S5, Supporting Information). The disappearance of ─CN stretching in dry samples indicated CH_3_CN dissociation. The absence of C─H stretching at 3053 cm^−1^ in Cu_14C‐S_ confirmed the attachment of sulfur atoms to the carbon atoms of *o*‐carborane, differing from that of Cu_14B‐S._

Single‐crystal X‐ray diffraction (SCXRD) analysis confirmed that the two isomeric Cu_14_ clusters possess a face‐centered cubic (FCC) metal framework, wherein a regular octahedral (oct) Cu_6_ core is embedded within a Cu_8_ cube (cub) (Figures S6 and S7, Supporting Information). Carboranedithiol ligand protected each cube face, adopting a consistent bridging mode (*μ_3_‐η^1^,η^2^*) linking with one Cu_oct_ and two Cu_cub_ atoms. CH_3_CN ligands were exclusively bound to the vertices of the cube. As shown in Figure S6 and Table S1 (Supporting Information), the Cu–Cu distance of the Cu_6_ kernel of Cu_14B‐S_ is longer than that of Cu_14C‐S_, while the bond length of cubic Cu_8_ shows the opposite tendency, suggesting a stronger core‐shell interaction in Cu_14B‐S_.

### 2.2 Hypergolic Performances of Isometric Cu_14_ Clusters

**Figure 2:**
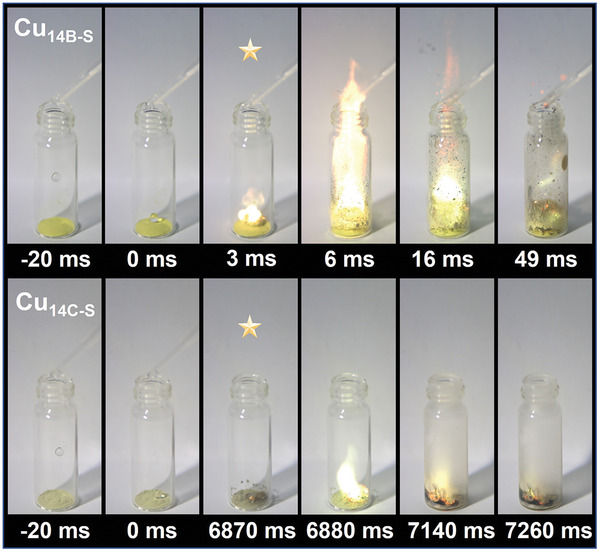
Hypergolicity drop tests with HTP for Cu_14B‐S_ (top) and Cu_14C‐S_ (bottom). Full videos for two of these processes are provided in the Supporting Information.

To assess the hypergolic properties of Cu_14B‐S_ and Cu_14C‐S_, various measurements were performed. The ID time, defined as the time between oxidizer contact and visible flame, was measured by the “oxidizer‐to‐fuel” droplet addition methodology and recorded by a high‐speed camera. As shown in **Figure** **2**, upon contact with HTP, Cu_14C‐S_ produced only white smog without a discernible flame for the first 100 ms. Its ID time was measured at a lengthy 6870 ms. In contrast, Cu_14B‐S_ exhibits exceptional performance with an ID time of 3 ms, rendering it among the top‐ranking H_2_O_2_ active hypergolic materials reported (**Table** **1**). It is worth noting that an ID time within 100 ms is an evaluation standard for acceptable and reliable rocket engine operation. Of note, the previously reported *o*‐carboranealkynyl‐protected [Cu_6_Ag_8_(C_4_B_10_H_11_)_12_(CH_3_CN)_2_]·2NO_3_ was sensitive to WFNA with an ID time of 15 ms but remained inert to HTP (Table 1; Figure S8, Supporting Information).^[^ ^29^ ^]^ Controlled experiments indicated that individual carboranyl ligands could not be ignited by oxidizers, highlighting the crucial role of the metal cluster core in catalyzing carborane ignition (Figure S9, Supporting Information). While a physical mixture of copper (I) salts and carboranyl ligands exhibited hypergolic behavior, probably due to the formation of a basic metal‐coordination complex, the ID time exceeded 12520 ms, limiting practical applications (Figure S10, Supporting Information). The aforementioned results demonstrated that the combination of carboranes with metal clusters could lead to potential new hypergolic properties, and the carborane derivatives could be regarded as versatile ligand platforms for fine‐tuning the catalytic effects of specific metal cluster cores.

**Table 1:** Hypergolic parameters of Cu_14B‐S_, Cu_14C‐S_, and the reported energetic materials.

Fuel	ID [ms]		Δ*H*_c_ [kJ mol^−1^]	*E*_g_ [kJ mol^−1^]	*E*_v_ [kJ mol^−1^]	*I*_SP_	FS [N]	IS [J]
	H_2_O_2_	HNO_3_						
Cu_14B‐S_	3	‐	−57925.6	24.4	34.0	217.7	120	>40
Cu_14C‐S_	6880	‐	−59683.6	24.3	27.1	217.2	40	>40
Cu‐PMIM‐1^[^ ^57^ ^]^	12	‐	‐	‐	‐	‐	>360	>40
[Cu(BTA)(NH_3_)_2_]_n_^[^ ^58^ ^]^	32	‐	−2540.0	‐	‐	250.0	‐	‐
Zn(ALM)_2_^[^ ^6^ ^]^	×	2	−4783.8	19.3	19.3	149.5	‐	‐
CBA‐CuAg^[^ ^29^ ^]^	×	15	−77355.0	‐	‐	‐	>360	>40
TNT^[^ ^59^ ^]^	×	×	−3439.0	15.4	26.2	‐	353	>40
CL‐20^[^ ^60^ ^]^	×	×	‐	6.3	‐	272.5	94	4

The heat of combustion (Δ*H*_c_) values for Cu_14B‐S_ and Cu_14C‐S_, determined by oxygen bomb calorimetry, were found to be −57925.6 kJ mol^−1^ and −59683.6 kJ mol^−1^, respectively (Table 1). These carborane‐based Cu_14_ compounds demonstrated favorable energy release characteristics compared to most hypergolic materials, attributed to the presence of abundant energy‐rich carboranes. The calculated gravimetric energy density (*E*_g_) and volumetric energy density (*E*_v_) based on Δ*H*_c_, along with the specific impulse (*I*_sp_) of the Cu_14_ clusters, were found to be comparable to or even higher than those of reported hypergolic fuels and conventional energetic compounds (TNT, CL‐20) (Table 1). Thermogravimetric analysis (TGA) showed that both Cu_14B‐S_ and Cu_14C‐S_ exhibit a continuous weight loss above 100 °C (Figure S11, Supporting Information). Furthermore, the safety parameters related to the impact and friction sensitivity of clusters were evaluated using the standard BAM drop hammer and friction tester technique. The impact sensitivity (IS) value of both clusters exceeds 40 J, and the friction sensitivity (FS) measurement indicates that Cu_14B‐S_ (120 N) is more stable than Cu_14C‐S_ (40 N). Taken together, these results highlight Cu_14B‐S_ as a promising candidate for hypergolic fuel, considering its comprehensive attributes of energy output and ignition performance.

## 3 Theoretical considerations

**Figure 3:**
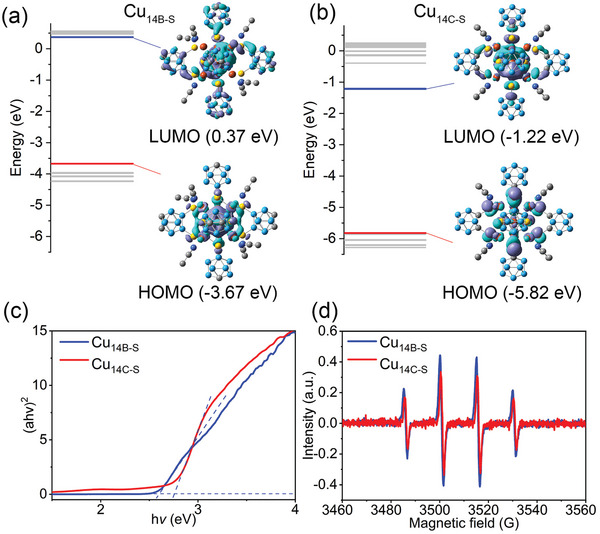
HOMO and LUMO and the corresponding calculated energy alignments of the a) Cu_14B‐S_ and b) Cu_14C‐S_. c) Tauc plots display the band gaps of Cu_14B‐S_ and Cu_14C‐S_. d) EPR spectra of the mixture of Cu_14_ and H_2_O_2_ in a ratio of 1:100.

To elucidate the distinct hypergolic behavior of these two copper‐cluster isomers, density functional theory (DFT) calculations were conducted_._ As shown in **Figure** **3**, despite both Cu_14B‐S_ and Cu_14C‐S_ isomers sharing identical metal core and valence electrons, their electronic structures are significantly different. The energy of the highest occupied molecular orbital (HOMO) in Cu_14B‐S_ is greater than that in Cu_14C‐S_. Moreover, the HOMO state of Cu_14B‐S_ is primarily governed by Cu atoms, whereas in Cu_14C‐S_, S atoms contribute significantly to the HOMO state. The enrichment of the contribution of the metal kernel to the HOMO likely imparts Cu_14B‐S_ with better hypergolic performance due to the cluster kernels serving as the catalytic center for the combustion of non‐hypergolic *o*‐carborane, and the ignition is a redox process which involving the electrons transfer from the HOMO of the cluster to the lowest unoccupied molecular orbital (LUMO) of H_2_O_2_. Following the geometry optimization of Cu_14C‐S_ and Cu_14B‐S_, the calculated energy gaps between HOMO and LUMO states are 4.04 eV and 4.60 eV, respectively. This trend in energy gap, as revealed by solid‐state UV–vis diffuse reflectance spectra, aligns with the DFT calculations (Figure 3c). The narrower energy gap of Cu_14B‐S_ implies higher sensitivity to the oxidizer.^[^ ^61^ ^]^

**Figure 4:**
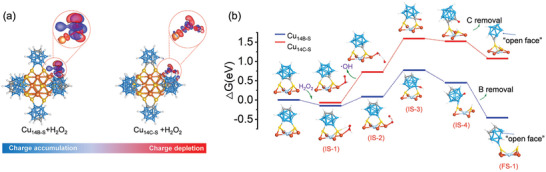
a) Differential charge density maps of Cu_14B‐S_ + H_2_O_2_ and Cu_14C‐S_ + H_2_O_2_. b) Optimized configurations of intermediates based on Cu_14B‐S_ and the calculated free energy diagram of Cu_14B‐S_ and Cu_14C‐S_ reacting with H_2_O_2_ molecule. For clarity, the calculated models are simplified, and the overall models are provided in Supporting Information. Color code: red, O.

Charge density difference calculation is a well‐established tool for analyzing the charge transfer between the catalyst and absorption molecule. Therefore, it was also utilized here to facilitate a more effective comparison of the nucleophilic activity between the two isomers. The differential charge density maps in **Figure** **4**a clearly indicate that Cu_14B‐S_ exhibits greater charge migration efficiency than Cu_14C‐S_. In other words, it will be more conducive to the charge transfer between the Cu_14B‐S_ and H_2_O_2_ molecules,^[^ ^62^ ^]^ which is consistent with the above experimental results. These distinctions could be attributed to the S atoms attaching to the distinct electronegativities of the B (electron donor) and C (electron acceptor) atoms, consequently modulating the charge distribution of the metal core.

### 3.1 Mechanistic Study of Hypergolicity with Hydrogen Peroxide Oxidizer

In general, the hypergolic ignition of fuel with HTP undergoes the exothermic catalytic dissociation of the H_2_O_2_. This process accompanies the release of high amounts of heat, resulting in the spontaneous ignition of the fuel–oxidizer mixture. To probe potential intermediates during the hypergolic reaction, electron paramagnetic resonance (EPR) spectra were performed with the mixture of H_2_O_2_ (30%) and Cu_14_ clusters. Employing 5,5‐dimethyl‐1‐pyrroline *n*‐oxide (DMPO) as a probe molecule, distinct signals corresponding to hydroxyl radicals (•OH) were readily detected for both clusters. The stronger intensity of Cu_14B‐S_ than that of Cu_14C‐S_ suggested its high activity toward H_2_O_2_ decomposition (Figure 3d). In addition, the removal of ligands plays a crucial role in enhancing cluster catalytic activity. As mentioned above, the auxiliary CH_3_CN ligands can readily detach from the Cu_14_ surface, thereby exposing the corresponding Cu atoms (Figure S5, Supporting Information).

On the basis of the above experimental and theoretical results, the partial hypergolic reaction pathways for the metal clusters ignited by H_2_O_2_ are proposed (Figure 4b; Figure S12 and Table S2, Supporting Information). First, H_2_O_2_ bonded with the vertex Cu atom, while the O─H···S hydrogen‐bond interaction between H_2_O_2_ and the cluster also facilitated their combination, giving the IS‐1 intermediates. The calculated energy barriers (ΔG) for this progress were −0.15 and −0.07 eV for Cu_14B‐S_ and Cu_14C‐S_, respectively. Second, under the catalysis of the metal kernel, the O─O bond of H_2_O_2_ was broken and formed •OH, yielding the intermediate state IS‐2. This process was highly endothermic and led to the intermediate‐state product including the Cu_14_ bonding with •OH, as well as dissociative •OH species. Compared with Cu_14C‐S_, Cu_14B‐S_ was more thermodynamically favorable for the catalytic decomposition of H_2_O_2_. Subsequently, benefitting from the steric effect and the shortest immigrant distance, the dissociative •OH tends to ligate on the adjacent B or C atom which bonded with the S atom (IS‐3). Whereafter, the B─S or C─S bond dissociation takes place, followed by the H bonded to the S (IS‐4). After that, the nucleophilic attack triggers the cleavage of the responding B or C from the carborane cluster. Compared to the removal of the C atom, the detachment of the B atom is extremely exothermic which is consistent with the experimental results. All of these results show that carboranyl ligands could effectively modulate the electronic structure of metal kernel, while the resultant metal kernel could regulate the hypergolic activity of carborane in turn.

## 4 Conclusion

In conclusion, this study delved into the hypergolic properties and ignition mechanism of two comparative examples of Cu_14_ cluster isomers when exposed to HTP. Despite their similar energetic attributes, Cu_14B‐S_ outshone Cu_14C‐S_ in terms of ignition delay time. Theoretical calculations indicated that Cu_14B‐S_ coupled with electron‐donating ligands could induce a narrower energy gap and more efficient charge migration, facilitating H_2_O_2_ absorption and activation, thereby expediting hypergolic behavior. A suggested reaction mechanism toward this hypergolic reaction was presented considering the active sites of the clusters and the generation of •OH of the mixture of Cu_14_ and H_2_O_2_. This study not only advances eco‐friendly hypergolic fuels without compromising performance but also provides important implications for the promotion of the hypergolic activity of the metal complex toward H_2_O_2_ ignition.

## Conflict of Interest

The authors declare no conflict of interest.

## Supporting information

Supporting Information

Supplemental Movie 1

Supplemental Movie 2

## Data Availability

The data that support the findings of this study are available from the corresponding author upon reasonable request.
