# Supplementary material for: Boosting 2000‐Fold Hypergolic Ignition Rate of Carborane by Substitutes Migration in Metal Clusters
Source: Adv Sci (Weinh). 2024 Apr 3;11(23):2401861. doi: 10.1002/advs.202401861 (PMC11186111; doi:10.1002/advs.202401861)
Supplement: Supplementary file 1 — Supporting Information [file ADVS-11-2401861-s003.docx]

Supporting Information

Boosting 2000-fold Hypergolic Ignition Rate of Carborane by Substitutes Migration in Metal Clusters

Jia-Hong Huang, Ao-Qi Ji, Zhao-Yang Wang, Qian-You Wang,* and Shuang-Quan Zang,*

J.-H. Huang, A.-Q. Ji, X.-Y. Dong, Z.-Y. Wang, Q.-Y. Wang, and S.-Q. Zang

Henan Key Laboratory of Crystalline Molecular Functional Materials and College of Chemistry, Zhengzhou University, Zhengzhou 450001 (China)
E-mail: [qianyouwang@zzu.edu.cn](mailto:qianyouwang@zzu.edu.cn) ; [zangsqzg@zzu.edu.cn](mailto:zangsqzg@zzu.edu.cn)

**Section S1: Materials and Methods**

**Materials and Reagents**

Tetrakis(acetonitrile)copper(I) hexafluorophosphate Cu(CH_3_CN)_4_PF_6_ and borane *tert*-butylamine were purchased from Aladdin. All reagents and solvents were used directly as received without further purification. 9,12-(HS)_2_-1,2-*closo*-carborane and 1,2-(HS)_2_-*closo*-carborane were prepared according to the literature method^1, 2^.

**Instrumentation.**

Powder X-ray diffraction (PXRD) patterns were collected using a Rigaku MiniFlex600 diffractometer (Cu Kα, *λ* = 1.54178 Å). EDS measurements were carried out using Zeiss Sigma 500. Fourier transform infrared (FT-IR) spectra were recorded in the range of 500-4000 cm^-1^ on a Bruker ALPHA II spectrometer. Ignition photos were captured using Phantom VEO710 high-speed charge-coupled device camera at 1000 frames/s. Bomb calorimetry was performed in a high-pressure bomb calorimeter (IDEA science BCA 500). In a typical experiment, 0.1 g of material was put into the crucible to be oxidized in the bomb under 30 bar of oxygen, and pellets of benzoic acid were used as a reference. Impact sensitivity was tested on the BAM Fall Hammer Impact Sensitivity Tester BFH 12 produced by OZM Research. Specific impulse data were calculated by means of NASA CEA software. Solid UV-Vis iffuse reflectance spectra were recorded with Hitachi UH4150 spectrometer. Electron paramagnetic resonance (EPR) spectroscopy was performed using a Bruker EMX plus 10/12 system.

**Hypergolic test.** Hypergolic test was evaluated by using standard oxidizer-to-fuel droplet tests. In a typical procedure, a single droplet of HTP (20 μL) was added to a 10 mL of glass vial containing a 10 mg sample. A high-speed camera operating at 1000 frames per seconds was used to record the ignition process. The ID time was measured between the first contact of the surface of the fuel with the oxidizer and the apparent visible flame. The tests were repeated several times. Full videos for these processes are provided in Video S1 and Video S2. The videos are playing for 200× slow motion.

**Caution!**

The energetic materials tend to explode under certain conditions. Thus, all the hypergolic testing in this study was performed in a fume hood, and anti-cutting gloves, leather coats, face shields, and earplugs were used.

**Section S2: Synthesis**

**Synthesis of Cu_14B-S_ and Cu_14C-S_**

Cu_14B-S_ and Cu_14B-S_ were prepared by a literature method with slight modifications^3, 4^. First, carboranedithiol ligands and Cu(CH_3_CN)_4_PF_6_ (15 mg, 0.055 mmol) were dissolved in 4 mL of acetonitrile. The solution turned clear within several minutes. To this solution, 6.0 mg of borane *tert*-butylamine (500 μL) was added under vigorous stirring. The resulting solution color changed from light yellow to dark yellow. Next, the yellow solution was filtered, and yellow crystals were formed within 5 days of evaporation. Collective yield Cu_14B-S_ and Cu_14C-S_ *approx*. 30.0% and 2%, respectively (based on Cu).

**Synthesis of [Cu_6_Ag_8_(C_4_B_10_H_11_)_12_(CH_3_CN)_2_]·2NO_3_**

[Cu_6_Ag_8_(C_4_B_10_H_11_)_12_(CH_3_CN)_2_]·2NO_3_ was prepared according to the literature method^5^.

**Section S3: Characterizations**


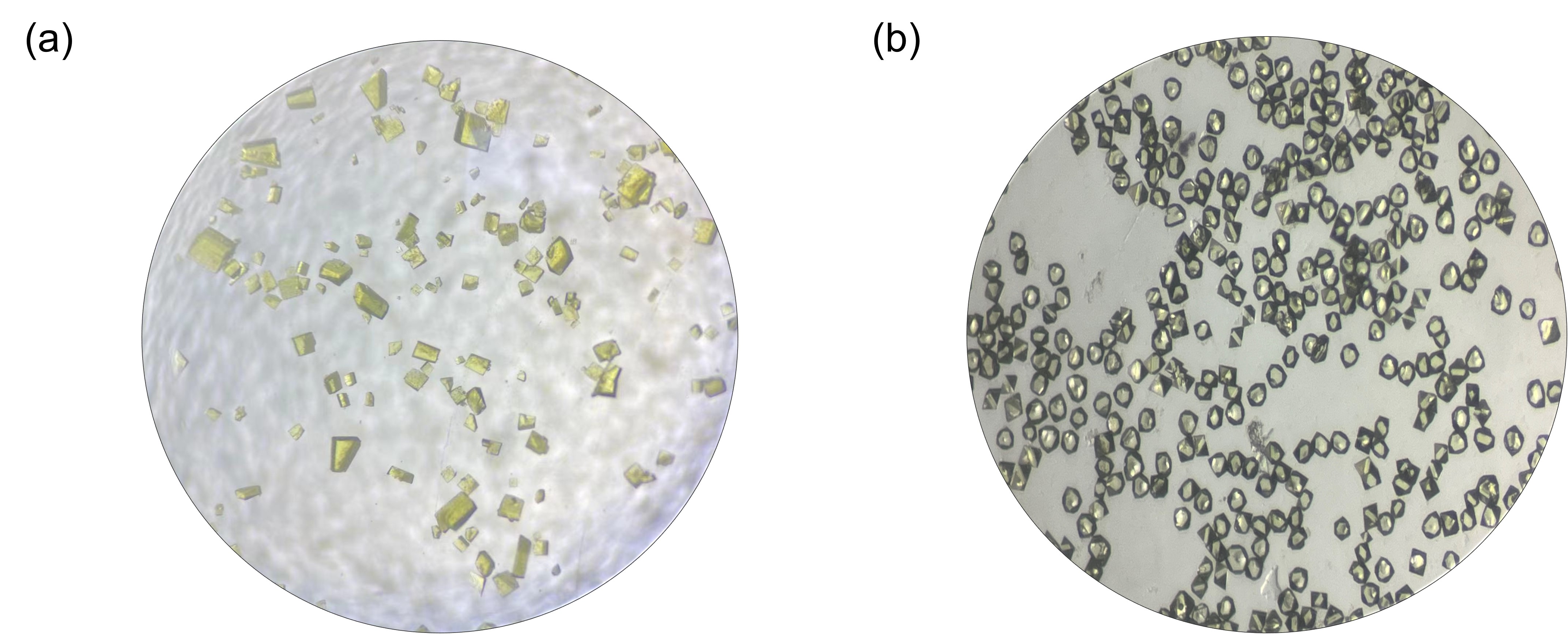


**Figure S1.** Images of Cu_14B-S_ (a) and Cu_14C-S_ (b) crystals.


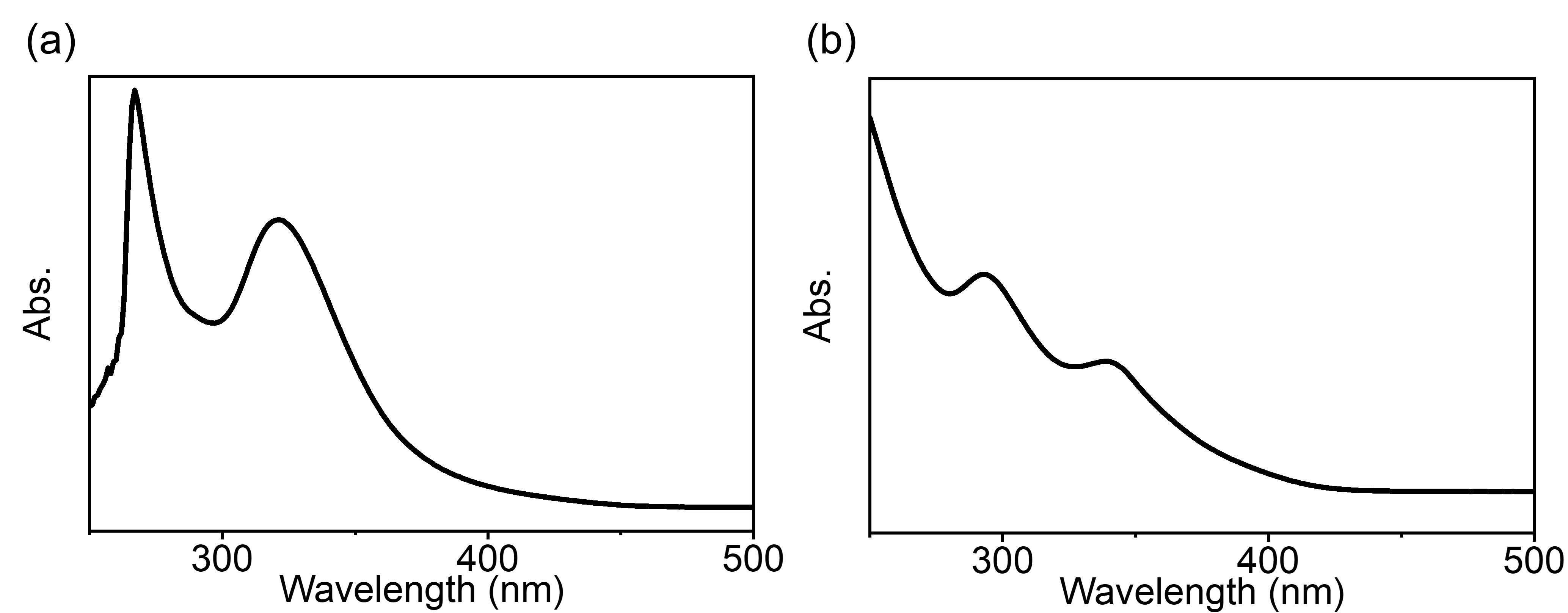


**Figure S2.** UV-vis absorption spectra of Cu_14B-S_ (a) and Cu_14C-S_ (b).


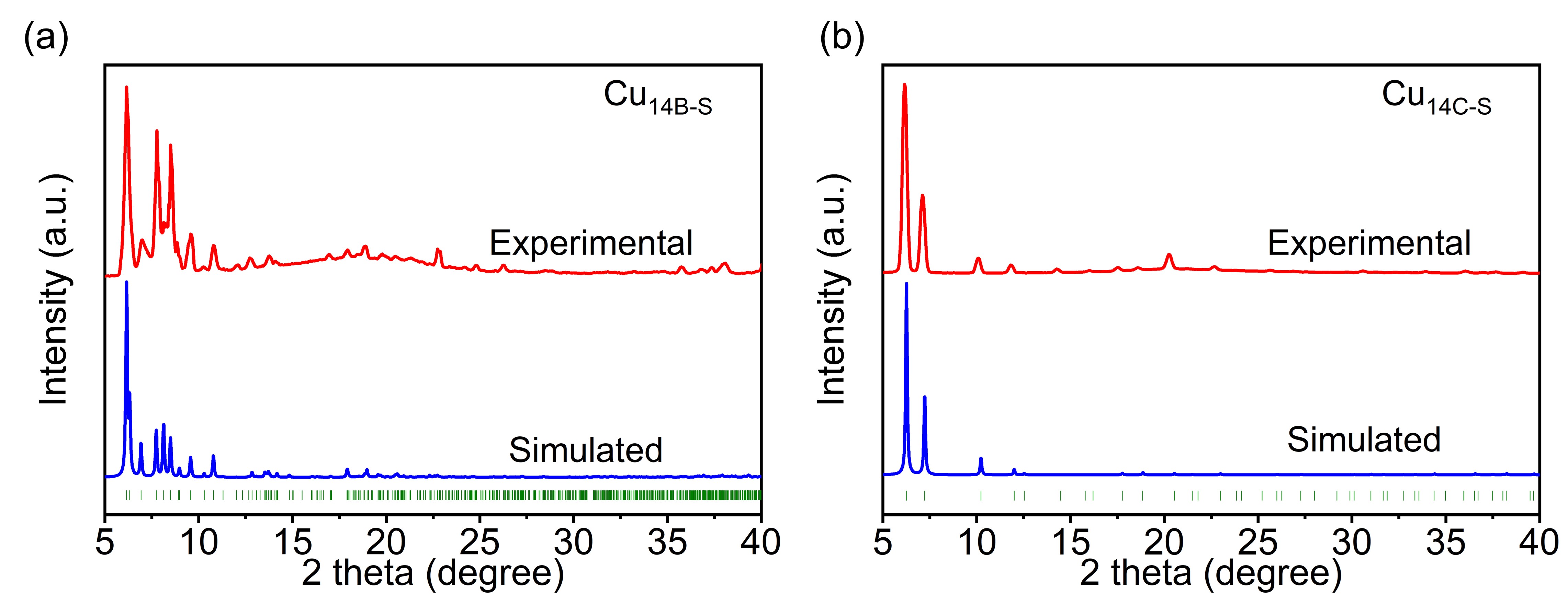


**Figure S3.** PXRD patterns of Cu_14B-S_ (a) and Cu_14C-S_ (b) crystals.


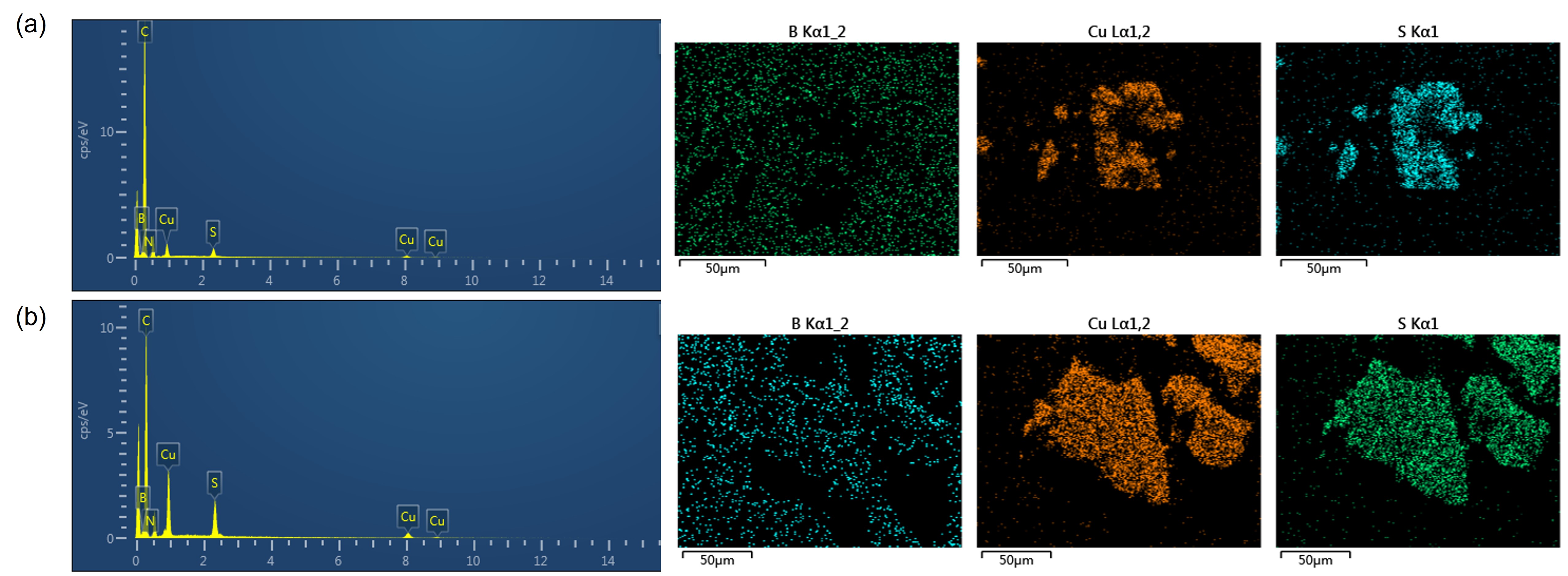


**Figure S4.** Energy dispersive spectroscopy of Cu_14B-S_ (a) and Cu_14C-S_ (b) crystals.


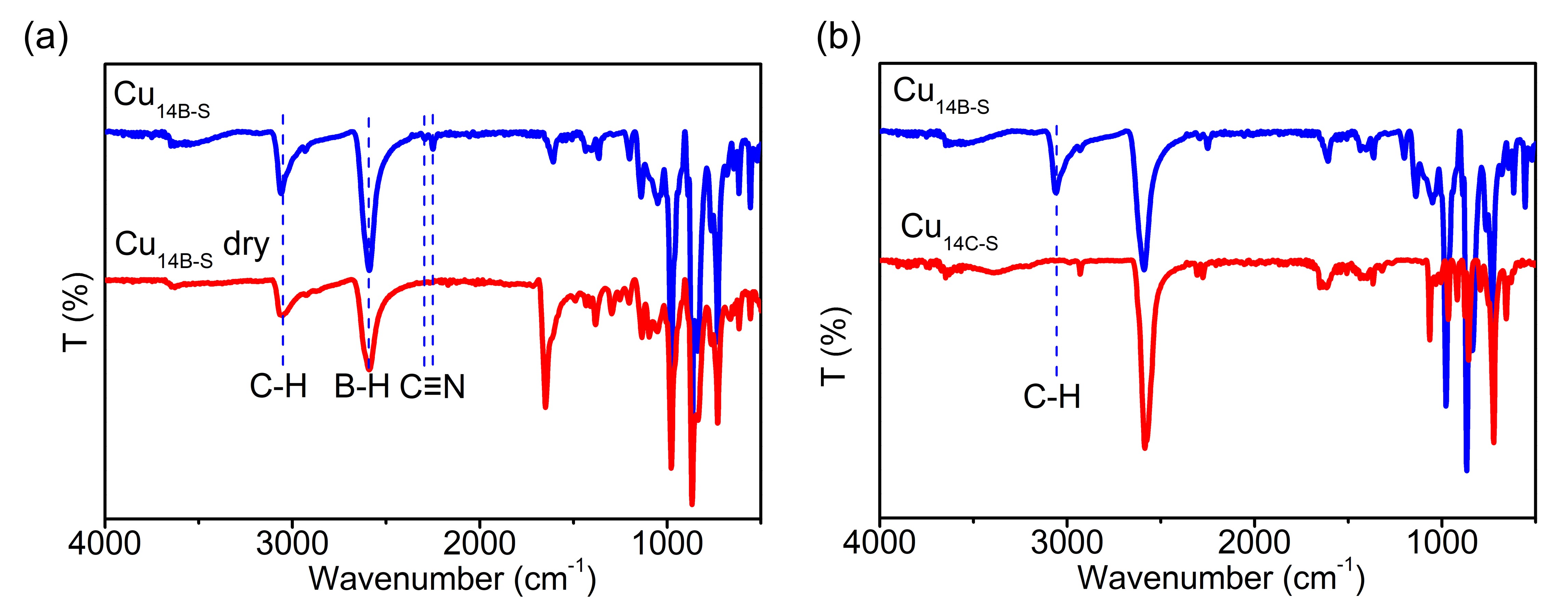


**Figure S5.** (a) FT-IR spectra of Cu_14B-S_ crystals before and after dry. (b) FT-IR spectra of Cu_14B-S_ and Cu_14C-S_.


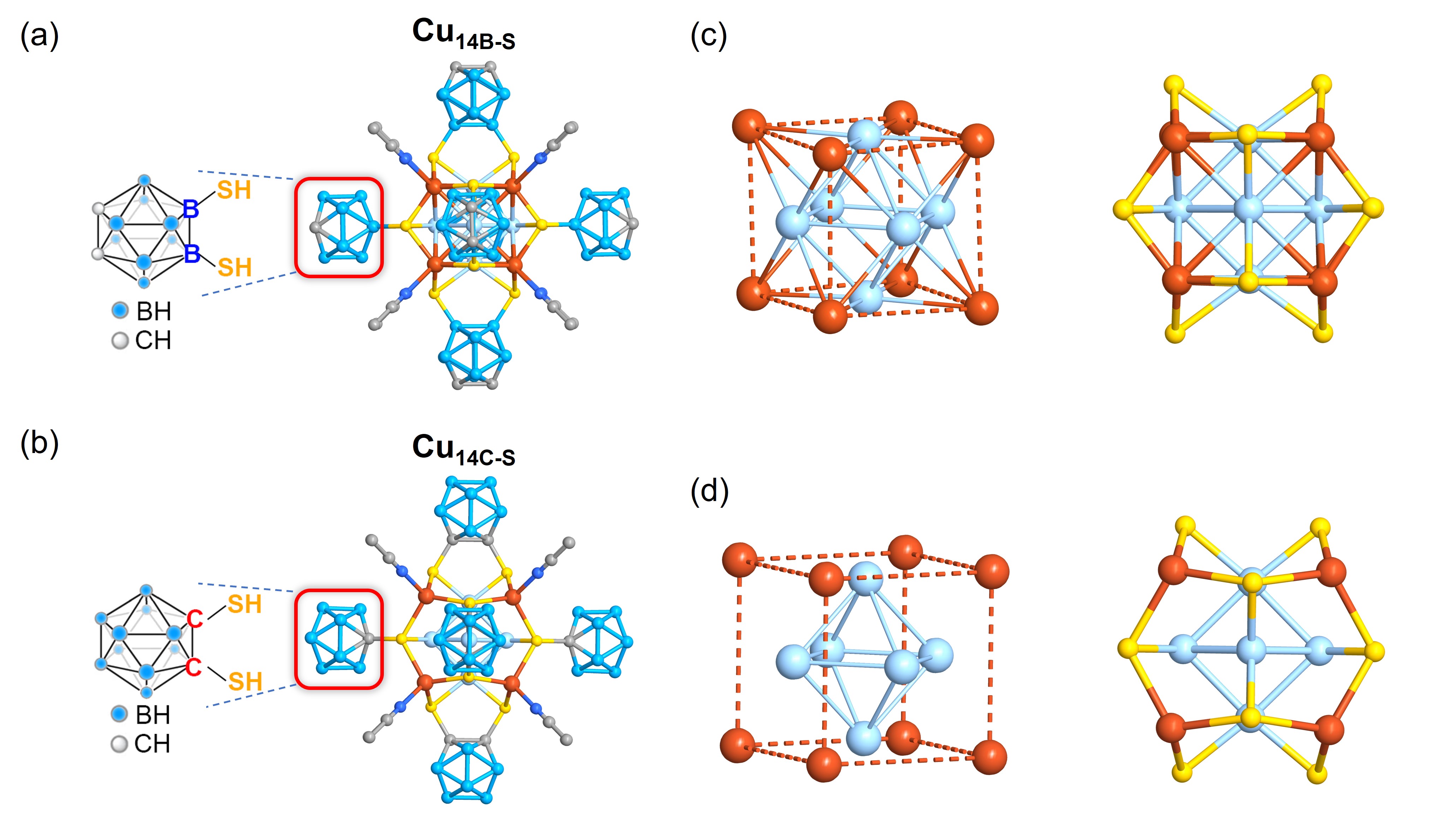


**Figure S6.** The overall structures of Cu_14B-S_ (a) and Cu_14C-S_ (b). Anatomy of the structures of Cu_14B-S_ (c) and Cu_14C-S_ (d). Color code: orange or light blue, Cu; yellow, S; blue, N; cyan, B; gray, C. For clarity, all H atoms are omitted.

_
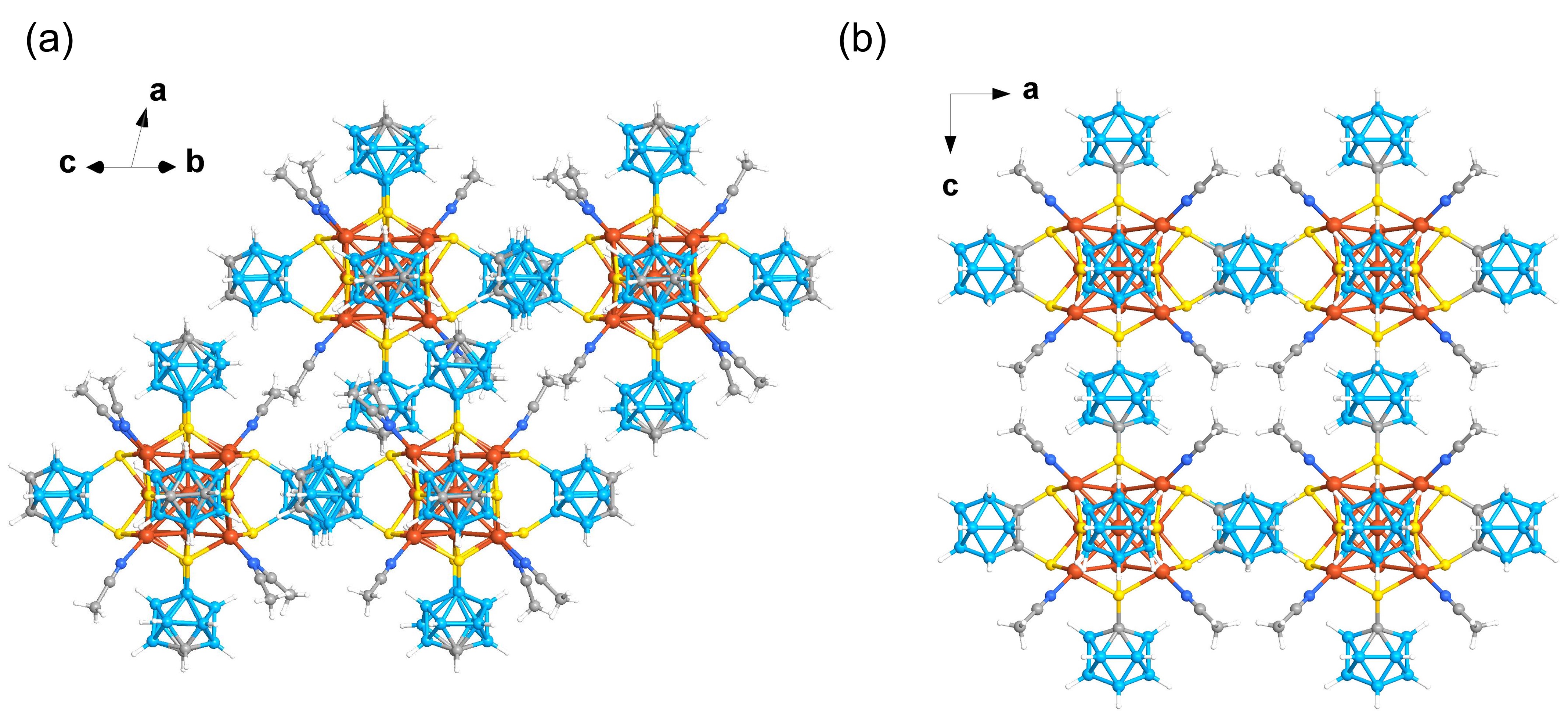
_

**Figure S7.** Packing structures of the Cu_14B-S_ (a) and Cu_14C-S_ (b). Color code: orange, Cu; yellow, S; blue, N; cyan, B; gray, C; white, H.


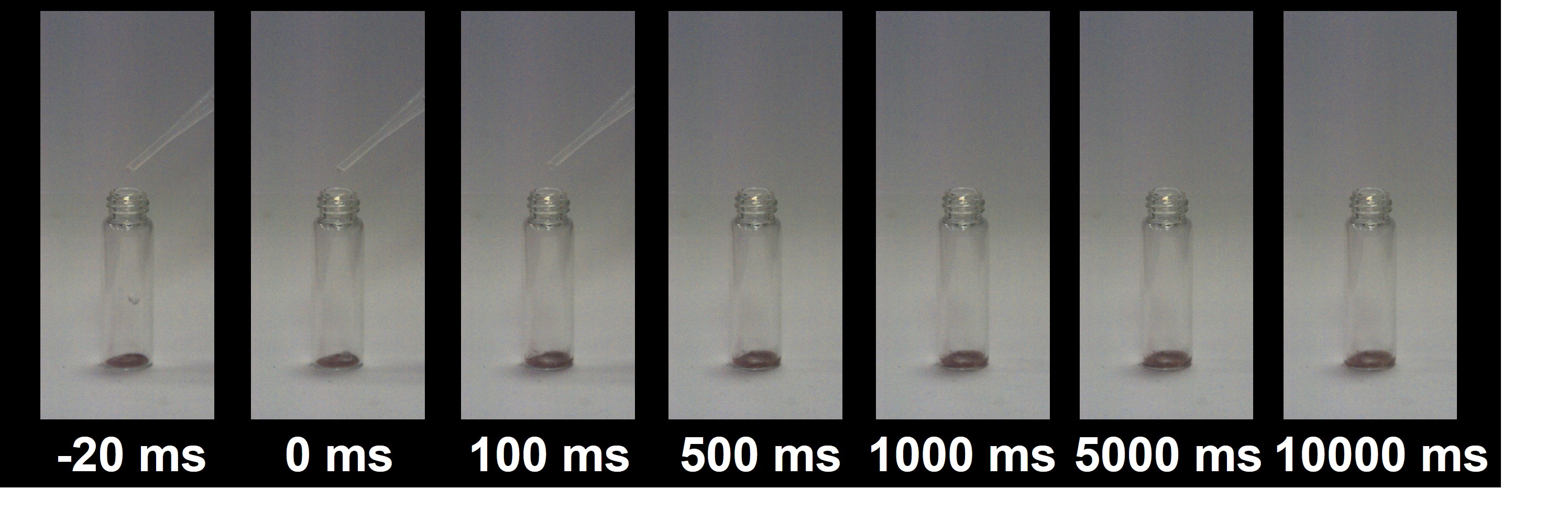


**Figure S8.** Hypergolicity drop tests with H_2_O_2_ for [Cu_6_Ag_8_(C_4_B_10_H_11_)_12_(CH_3_CN)_2_]·2NO_3_.

_
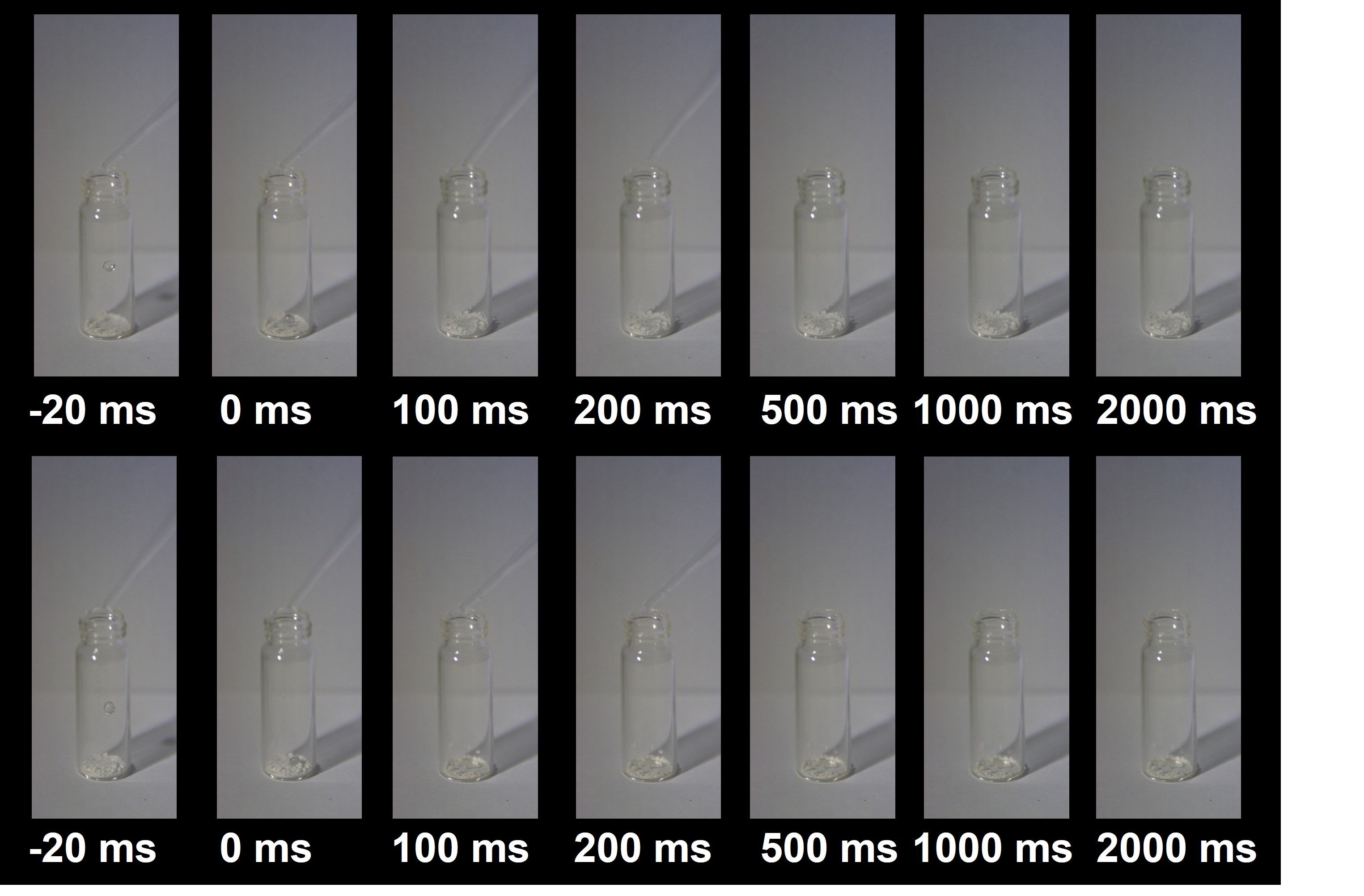
_

**Figure S9.** Hypergolicity drop tests with H_2_O_2_ for 9,12-(HS)_2_-1,2-*closo*-carborane (top) and 1,2-(HS)_2_-*closo*-carborane (bottom).


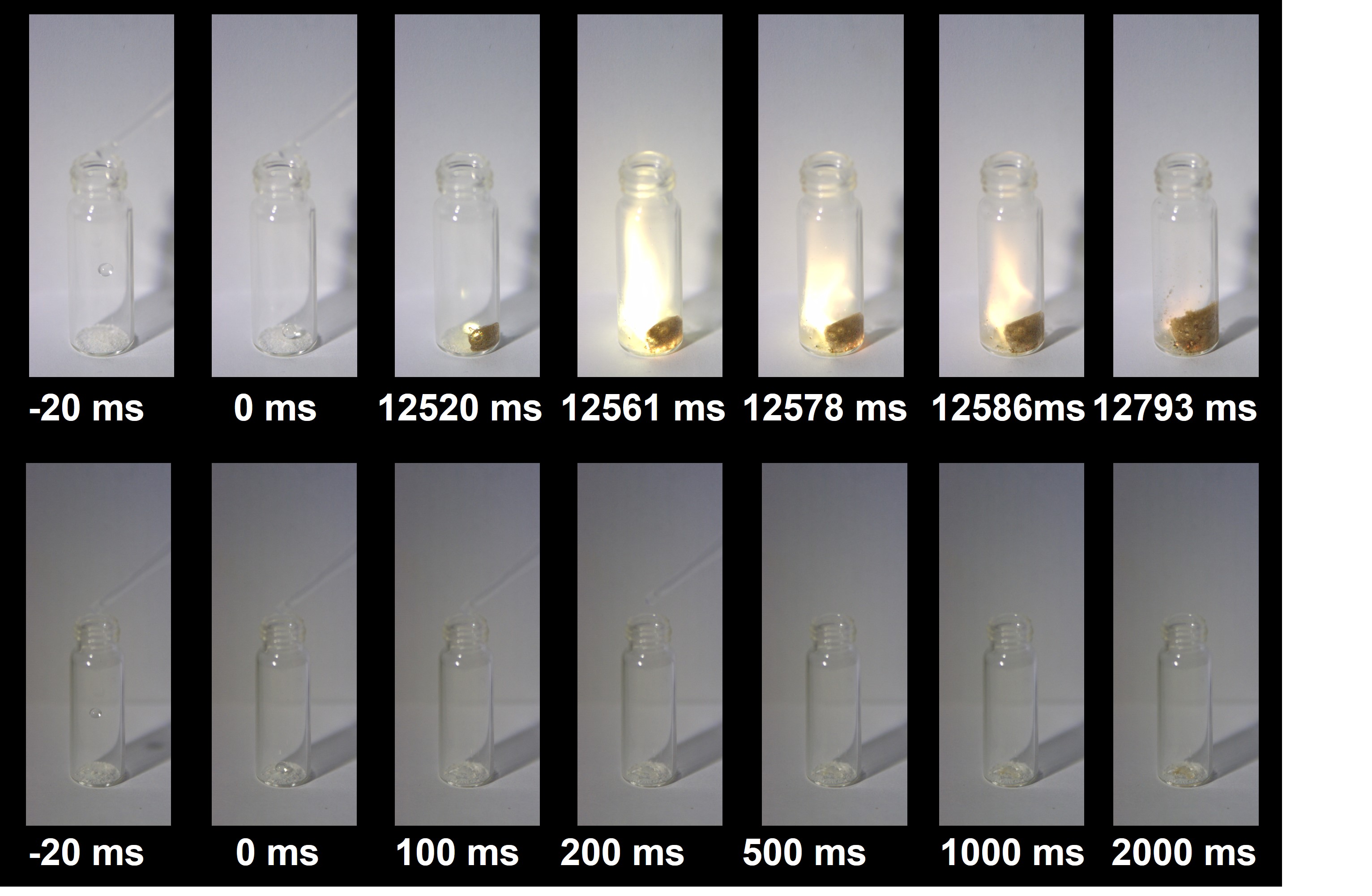


**Figure S10.** Hypergolicity drop tests with H_2_O_2_ for the mixture of 9,12-(HS)_2_-1,2-*closo*-carborane and Cu(CH_3_CN)_4_PF_6_ (top), and 1,2-(HS)_2_-*closo*-carborane and Cu(CH_3_CN)_4_PF_6_ (bottom).

**
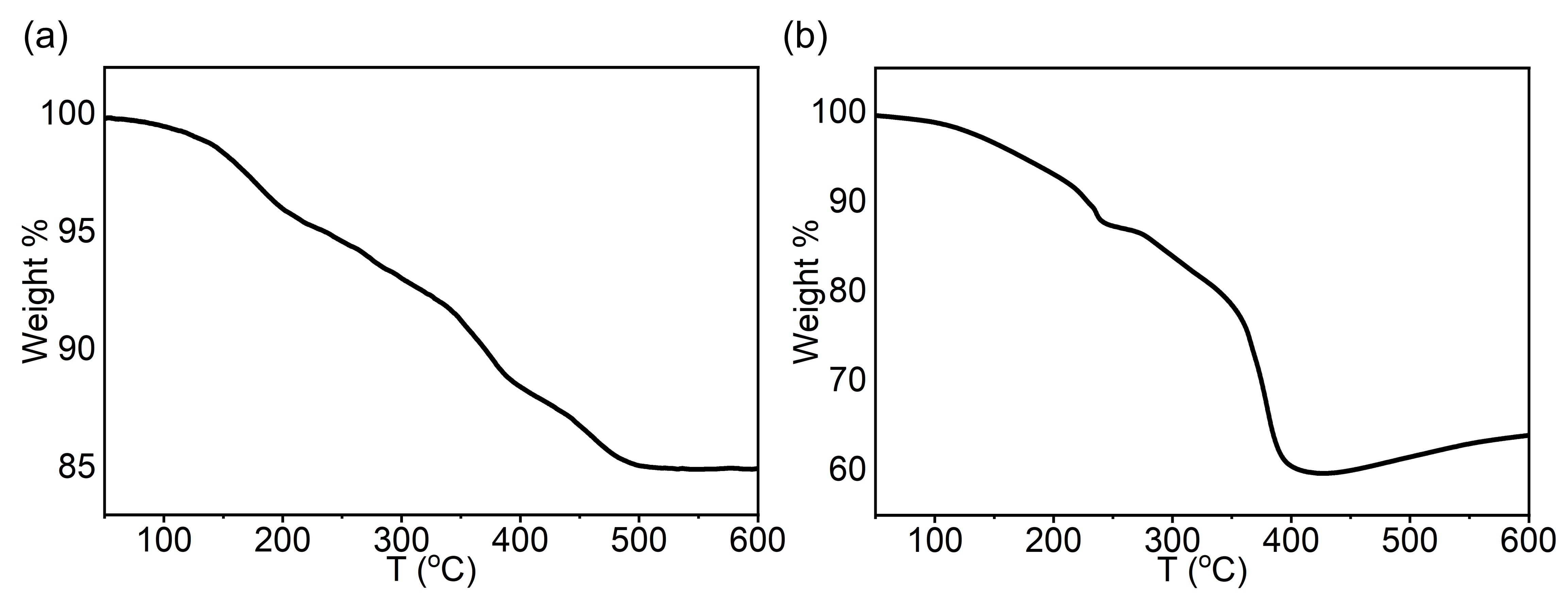
**

**Figure S11.** TGA curves of Cu_14B-S_ (a) and Cu_14C-S_ crystals (b).

**Section S4 Combustion Calorimetry**

**Calculation of specific impulse (*I*_sp_)**

The specific impulse of metal clusters was calculated using the program of NASA Chemical Equilibrium with Applications (CEA) code, according to the equation *I*_sp_ = k$\sqrt{Tc/M}$. In the whole calculation, the following parameters are considered: ambient temperature = 298.15 K, Pc = 25 atm, Pe = 1 atm, Ae/At = 4, and freezing flow conditions during expansion. The variation of O/F ranges from 1 to 5. The oxidant is HTP, which is consistent with the oxidant used in the subsequent hypergolic test.

**The possible products resulting from the oxidation of** **carborane by H_2_O_2_.**

Since the combustion reaction between carborane cluster and H_2_O_2_ occurred transiently, the intermediate products can’t be detected. Referring to the previous reports, the oxidation of carborane by H_2_O_2_ can induce partial degradation, thence conversion of the *closo*-C_2_B_10_ to the *nido*-[C_2_B_9_]^-^ species.^6^ In addition, it would probably lead to a variety of possible intermediate products, including C_2_B_9_H_10_, C_2_B_9_H_11_, C_2_B_9_H_11_^+^, CB_9_H_9_, CB_9_H_9_^+^, CB_10_H_11_, CB_10_H_11_^+^, and C_2_B_10_H_12_^+^, by removing CH, CH^+^, BH, BH^+^, BH_2_, BH_2_^+^, H_2_, H, H^+^, BCH_3_, and BCH_3_^+^.^7^ The final combustion product is B_2_O_3_.

**Section S5 Theoretical calculations**

**Calculations of the theoretical structure of Cu_14B-S_ and Cu_14C-S_**

To gain insight into the effects of the electronic structure, the density functional theory (DFT) on the clusters is performed based on their single-crystal structures (Figure 1). DFT calculation was performed with Gaussian 16^8^ under the Perdew−Burke−Ernzerhof (PBE) exchange-correlation functional^9^ using def2SVP^10^ basis set for all atoms. The single-crystal structure was chosen as the initial guess for ground-state optimization, and all reported stationary points were verified as true minima by the absence of negative eigenvalues in the vibrational frequency analysis.

**Differential charge density maps and the catalytic reactions of H_2_O_2_ and clusters.**

All the calculations were performed within the framework of the DFT as implemented in the Vienna Ab initio Software Package (VASP 5.4.4) code within the Perdew–Burke–Ernzerhof (PBE) generalized gradient approximation and the projected augmented wave (PAW) method^11^. The cutoff energy for the plane-wave basis set was set to 450 eV. The Brillouin zone of the surface unit cell was sampled by Monkhorst–Pack (MP) grids, with k-point mesh density of 2π × 0.04 Å^-1^ for structures optimizations^12^. The convergence criterion for the electronic self-consistent iteration and force was set to 10−5 eV and 0.01 eV/Å, respectively. The vacuum layer of 15 Å was introduced to avoid interactions between periodic images.


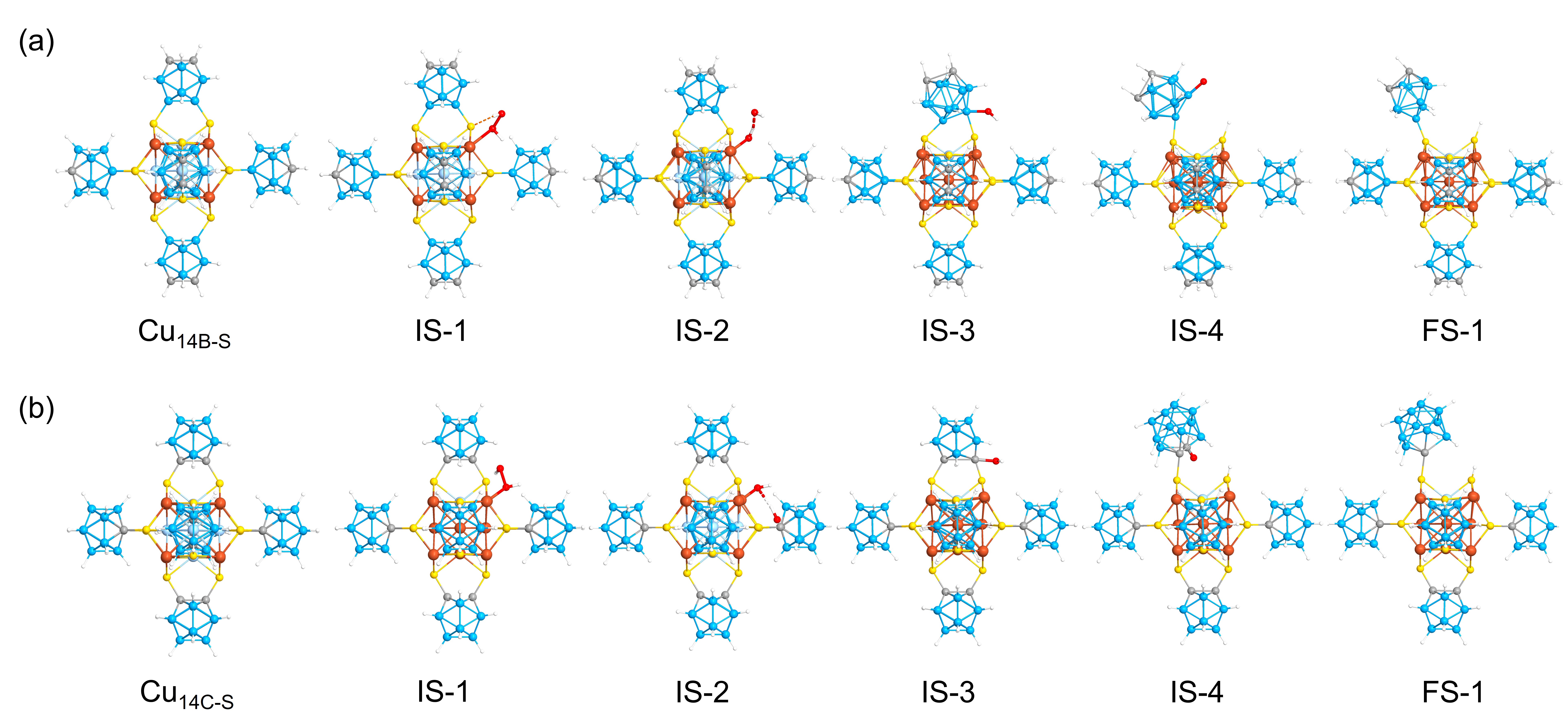


**Figure S12.** Optimized configurations of intermediates based on Cu_14B-S_ (a) and Cu_14C-S_ (b)_._ Color code: orange or light blue, Cu; yellow, S; red, O; blue, N; cyan, B; gray, C; white, H.

**Section S6 Supporting Tables**

**Table S1.** Comparison of bond lengths in Cu_14B-S_ and Cu_14C-S_ clusters.


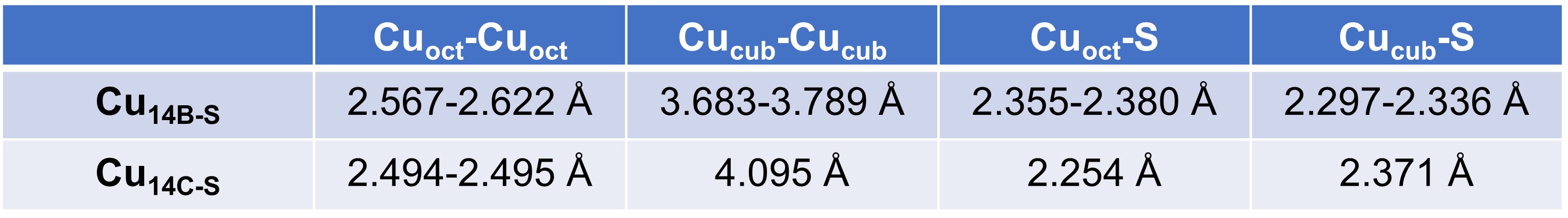


**Table S2.** Calculated free energy results of the transition states of Cu_14B-S_ and Cu_14C-S_ reacting with the H_2_O_2_ molecule.


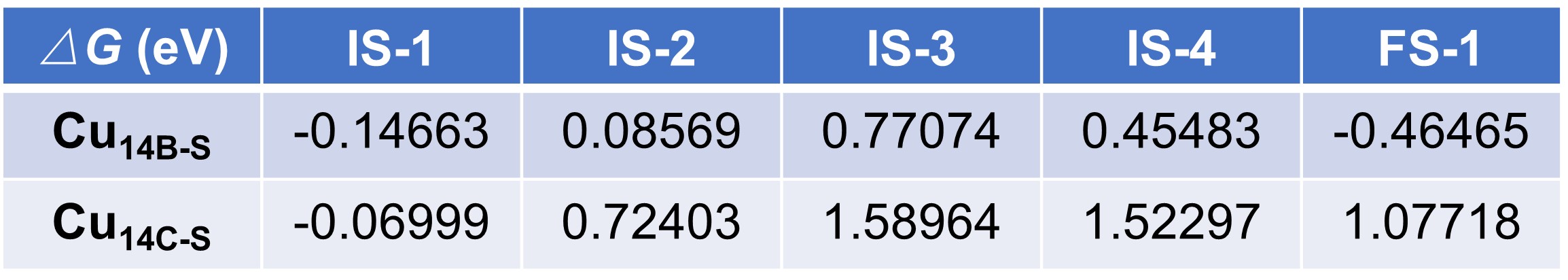


**Table S3.** Crystal and structure determination data.^3, 4^

| Identification coded | **Cu_14B-S_** | **Cu_14C-S_** |
| --- | --- | --- |
| CCDC number | 2177976 | 1861371 |
| Empirical formula | C_24_H_78_B_60_Cu_14_N_6_S_12_ | C_24_H_84_B_60_Cu_14_N_8_S_12_ |
| Formula weight | 2373.80 | 2456.06 |
| Temperature/K | 200.00 | 200.00 |
| Crystal system | Monoclinic | Cubic |
| Space group | *C*2/*c* | *Fm*-3*m* |
| *a*/Å | 22.0785(5) | 24.4525(2) |
| *b*/Å | 19.8035(4) | 24.4525(2) |
| *c*/Å | 27.0857(4) | 24.4525(2) |
| *α*/° | 90.0 | 90.0 |
| *β*/° | 109.7 | 90.0 |
| *γ*/° | 90.0 | 90.0 |
| Volume/Å^3^ | 11147.9(4) | 14620.7(3) |
| Z | 4 | 4 |
| ρ_calc_g/cm^3^ | 1.414 | 1.116 |
| *μ*/mm^‑1^ | 5.094 | 3.903 |
| *F*(000) | 4684.0 | 4824.0 |
| Crystal size/mm^3^ | 0.02 × 0.02 × 0.02 | 0.14 ×0.06 ×0.04 |
| Radiation | Cu *Kα* (*λ* = 1.54184) | Cu *Kα* (*λ* = 1.54184) |
| 2*θ* range for data collection/° | 6.164 to 148.49 | 6.26 to 134.562 |
| Index ranges | -27 ≤ *h* ≤ 20,  -23 ≤ *k* ≤ 24,  -33≤ *l* ≤ 33 | -16≤ *h* ≤ 18,  -26 ≤ *k* ≤ 19,  -29 ≤ *l* ≤ 0 |
| Reflections collected | 29358 | 3273 |
| Independent reflections | 11018 [*R_int_* = 0.0352, *R_sigma_* = 0.0421] | 10097 [*R_int_* = 0.0209, *R_sigma_* = 0.0229] |
| Data/restraints/parameters | 11018/253/904 | 710/7/55 |
| Goodness-of-fit on *F^2^* | 1.052 | 1.182 |
| Final R indexes [I>=2σ (I)] | *R_1_* = 0.0925,  *wR_2_* = 0.2677 | *R_1_* = 0.0400  *wR_2_* = 0.1459 |
| Final R indexes [all data] | *R_1_* = 0.1040,  *wR_2_* = 0.2766 | *R_1_* = 0.0488  *wR_2_* = 0.1551 |
| Largest diff. peak/hole / e Å^-3^ | 1.59/-0.90 | 0.34/-0.96 |

**Reference:**

[1] Plešek, J.; Janoušek, Z.; Heřmánek, S. *Collect. Czech. Chem. Commun.* **1980**, *45*, 1775.

[2] Viñas, C.; Benakki, R.; Teixidor, F.; Casabó, J. *Inorg. Chem.***1995**, *34*, 3844-3845.

[3] Huang, J.-H.; Liu, L.-Y.; Wang, Z.-Y.; Zang, S.-Q.; Mak, T. C. W., Modular Cocrystallization of Customized Carboranylthiolate-Protected Copper Nanoclusters via Host-Guest Interactions. *ACS Nano* **2022**, *16*, 18789-18794.

[4] Li, Y.-L.; Wang, J.; Luo, P.; Ma, X.-H.; Dong, X.-Y.; Wang, Z.-Y.; Du, C.-X.; Zang, S.-Q.; Mak, T. C. W., Cu_14_ Cluster with Partial Cu(0) Character: Difference in Electronic Structure from Isostructural Silver Analog. *Adv. Sci.* **2019**, *6*, 1900833.

[5] Wang, Q.-Y.; Wang, J.; Wang, S.; Wang, Z.-Y.; Cao, M.; He, C.-L.; Yang, J.-Q.; Zang, S.-Q.; Mak, T. C. W., *o*-Carborane-Based and Atomically Precise Metal Clusters as Hypergolic Materials. *J. Am. Chem. Soc.* **2020**, *142*, 12010-12014.

[6] C. Viñas, R. Núñez, I. Rojo, F. Teixidor, R. Kivekäs, and R. Sillanpää, Proton Mediated Partial Degradation of *Closo*-dicarbaboranes. *Inorg. Chem.* **2001**, *40*, 3259-3260.

[7] D. Feng, J. Liu, A. P. Hitchcock, A. L. Kilcoyne, T. Tyliszczak, N. F. Riehs, E. Ruhl, J. D. Bozek, D. McIlroy, P. A. Dowben, *J. Phys. Chem. A* **2008**, *112*, 3311.

[8] M. J. Frisch, *et al*. Gaussian, Inc., Wallingford CT, **2016**.

[9] J. P. Perdew, K. Burke, M. Ernzerhof, *Phys. Rev. Lett*. **1996**, *77*, 3865.

[10] (a) Pritchard, Benjamin P., Altarawy, Doaa, Didier, Brett, Gibsom, Tara D., Windus, Theresa L. A New Basis Set Exchange: An Open, Up-to-date Resource for the Molecular Sciences Community. *J. Chem. Inf. Model.* **2019**, *59*, 4814. (b) Feller, David. The role of databases in support of computational chemistry calculations. *J. Comput. Chem.* **1996**, *17*, 1571. (c) Schuchardt, Karen L., Didier, Brett T., Elsethagen, Todd, Sun, Lisong, Gurumoorthi, Vidhya, Chase, Jared, Li, Jun, Windus, Theresa L. Basis Set Exchange: A Community Database for Computational Sciences. *J. Chem. Inf. Model.* **2007**, *47*, 1045. (d) Andrae, D., U. Häußermann, U., Dolg, M., Stoll, H., H. Preuß, H. Energy-adjusted ab initio pseudopotentials for the second and third row transition elements. *Theor. Chim. Acta* **1990**, *77*, 123. (e) Weigend, Florian, Ahlrichs, Reinhart. Balanced basis sets of split valence, triple zeta valence and quadruple zeta valence quality for H to Rn: Design and assessment of accuracy. *Phys. Chem. Chem. Phys.* **2005**, *7*, 3297.

[11] (a) Perdew, J. P.; Burke, K.; Ernzerhof, M., Generalized Gradient Approximation Made Simple. *Phys. Rev. Lett*. **1996**, *77*, 3865. (b) Hammer, B.; Hansen, L. B.; Nørskov, J. K., Improved adsorption energetics within density-functional theory using revised Perdew-Burke-Ernzerhof functionals. *Phys. Rev. B* **1999**, *59*, 7413. (c) Blöchl, P. E., Projector augmented-wave method. *Phys. Rev. B* **1994**, *50*, 17953. (d) Kresse, G.; Joubert, D., From ultrasoft pseudopotentials to the projector augmented-wave method. *Phys. Rev. B* **1999**, *59*, 1758.

[12] Monkhorst, H. J.; Pack, J. D., Special points for Brillouin-zone integrations. *Phys. Rev. B* **1976**, *13*, 5188.
